# Supplementary material for: Trophy hunters pay more to target larger-bodied carnivores
Source: R Soc Open Sci. 2019 Sep 18;6(9):191231. doi: 10.1098/rsos.191231 (PMC6774968; doi:10.1098/rsos.191231)
Supplement: Figure S2 from Trophy hunters pay more to target larger-bodied carnivores [file rsos191231supp2.docx]

**Figure S2. (a)** Model-averaged and **(b)** global model predictions for the presence and absence of a ‘dangerous’ and/or ‘difficult’ description included in the Safari Club International (SCI) online record book hunt remarks section. Predictions include mean values for conservation status and mass terms. Points indicate the predictions for expected mean prices for each classification (carnivore or ungulate). Lines indicate the 95% confidence level.

**
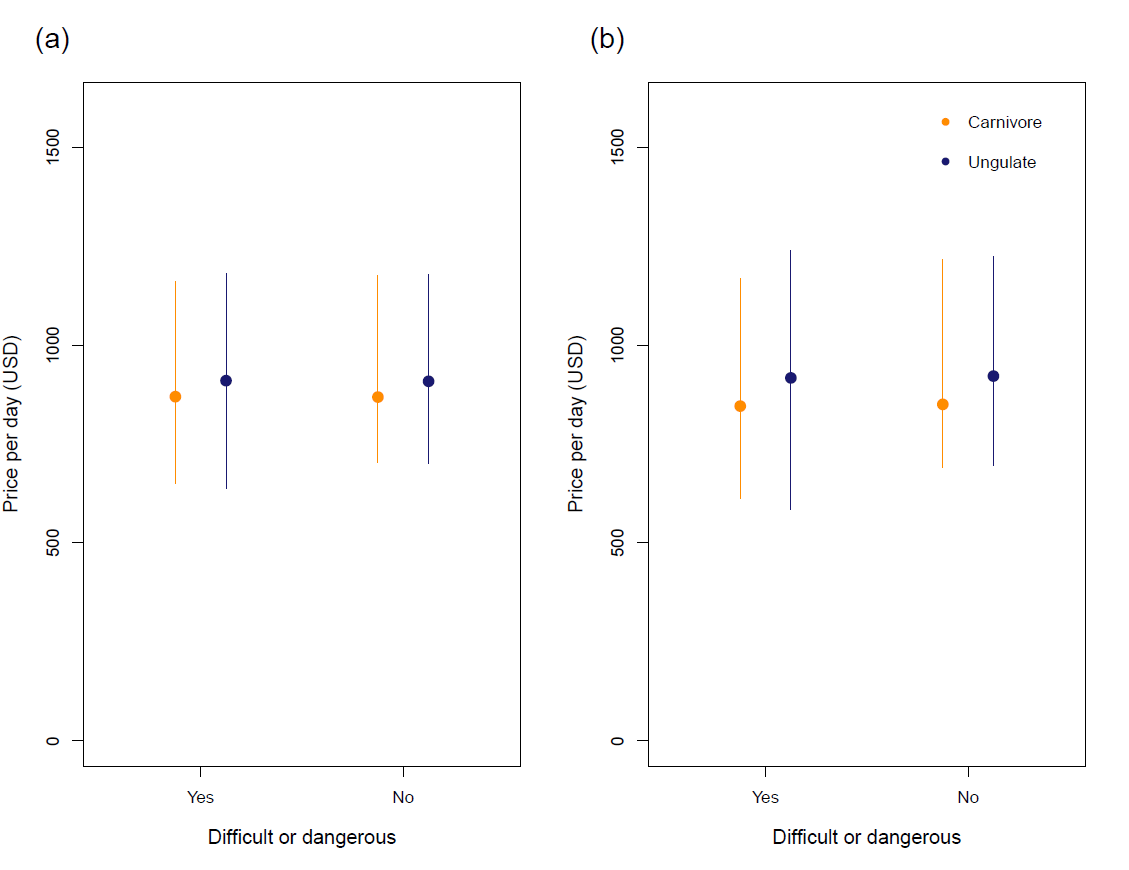
**
