# Supplementary material for: Trophy hunters pay more to target larger-bodied carnivores
Source: R Soc Open Sci. 2019 Sep 18;6(9):191231. doi: 10.1098/rsos.191231 (PMC6774968; doi:10.1098/rsos.191231)

**Figure S3.** (**a)** Model-averaged and **(b)** global model predictions (solid-lines) for the effect of conservation status on price for carnivore (orange) and ungulate (blue) species. Predictions include mean values for mass and Safari Club International (SCI) terms. Points show raw status data for carnivores and ungulates. Shading indicates 95% confidence levels for predictions.


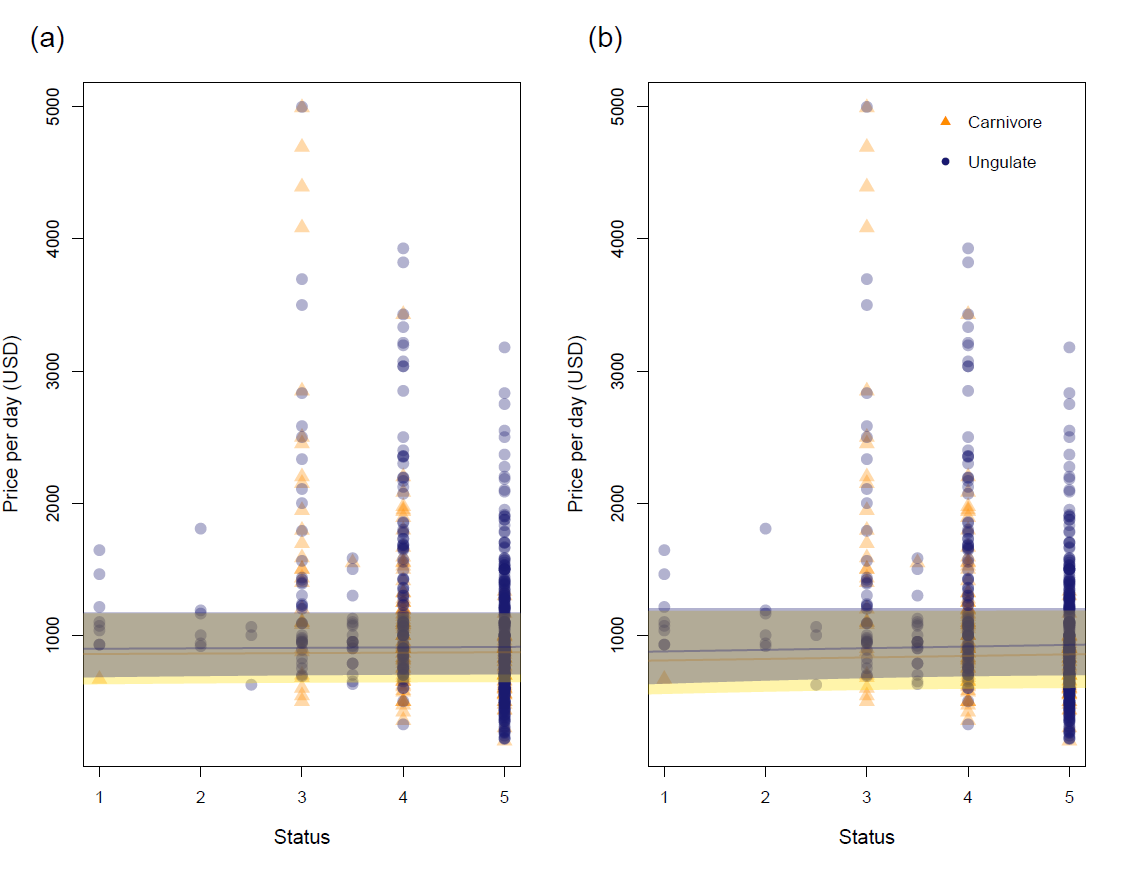

Supplement: Figure S3 from Trophy hunters pay more to target larger-bodied carnivores [file rsos191231supp3.docx]
