# Supplementary material for: Trophy hunters pay more to target larger-bodied carnivores
Source: R Soc Open Sci. 2019 Sep 18;6(9):191231. doi: 10.1098/rsos.191231 (PMC6774968; doi:10.1098/rsos.191231)

**Figure S4.** Predictions (solid line) for the relationship between latitude (decimal degrees; calculated by using the average latitude for each jurisdiction) and mass, for carnivore (orange) and ungulate (blue) species. Points show raw mass data. Shading indicates 95% confidence levels for predictions.


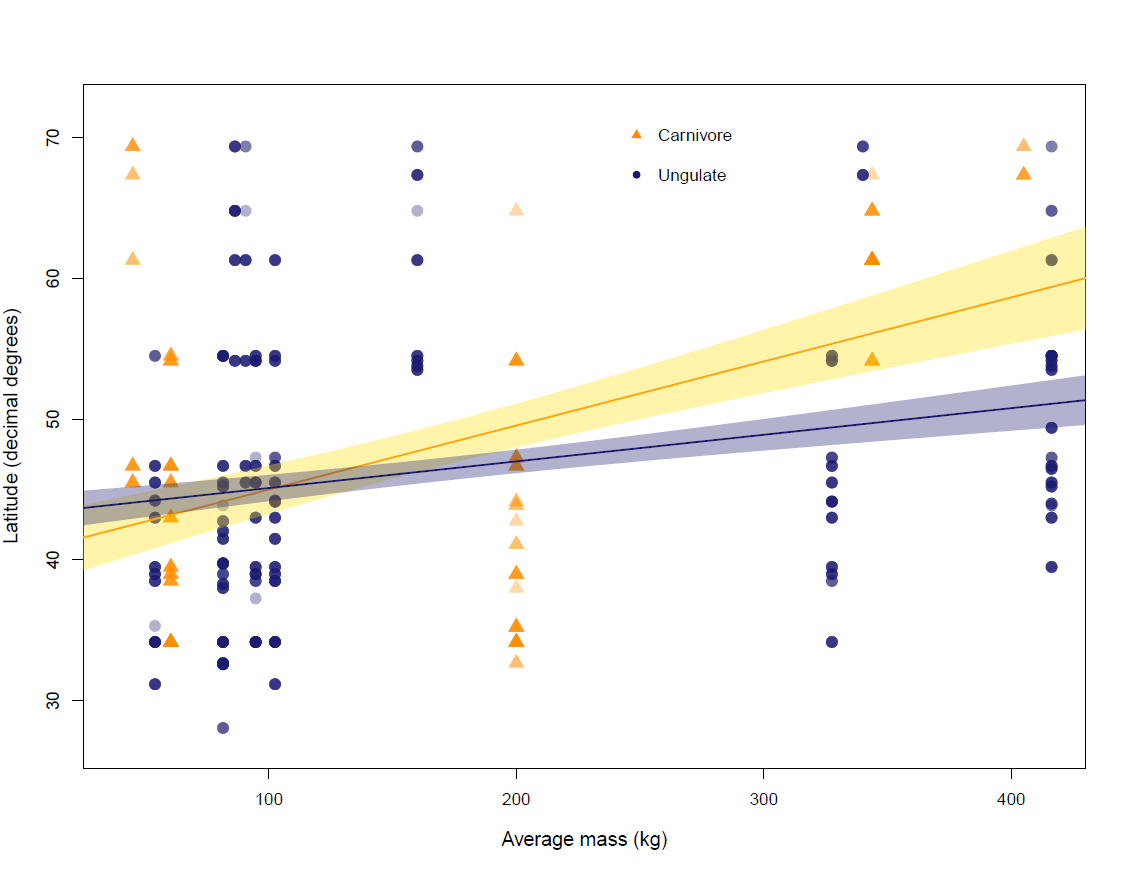

Supplement: Figure S4 from Trophy hunters pay more to target larger-bodied carnivores [file rsos191231supp4.docx]
