# Supplementary material for: Trophy hunters pay more to target larger-bodied carnivores
Source: R Soc Open Sci. 2019 Sep 18;6(9):191231. doi: 10.1098/rsos.191231 (PMC6774968; doi:10.1098/rsos.191231)

**Figure S5.** Top model (Table 2) with latitude replacing mass, suggesting latitude does not have the same relationship with guide price as mass.


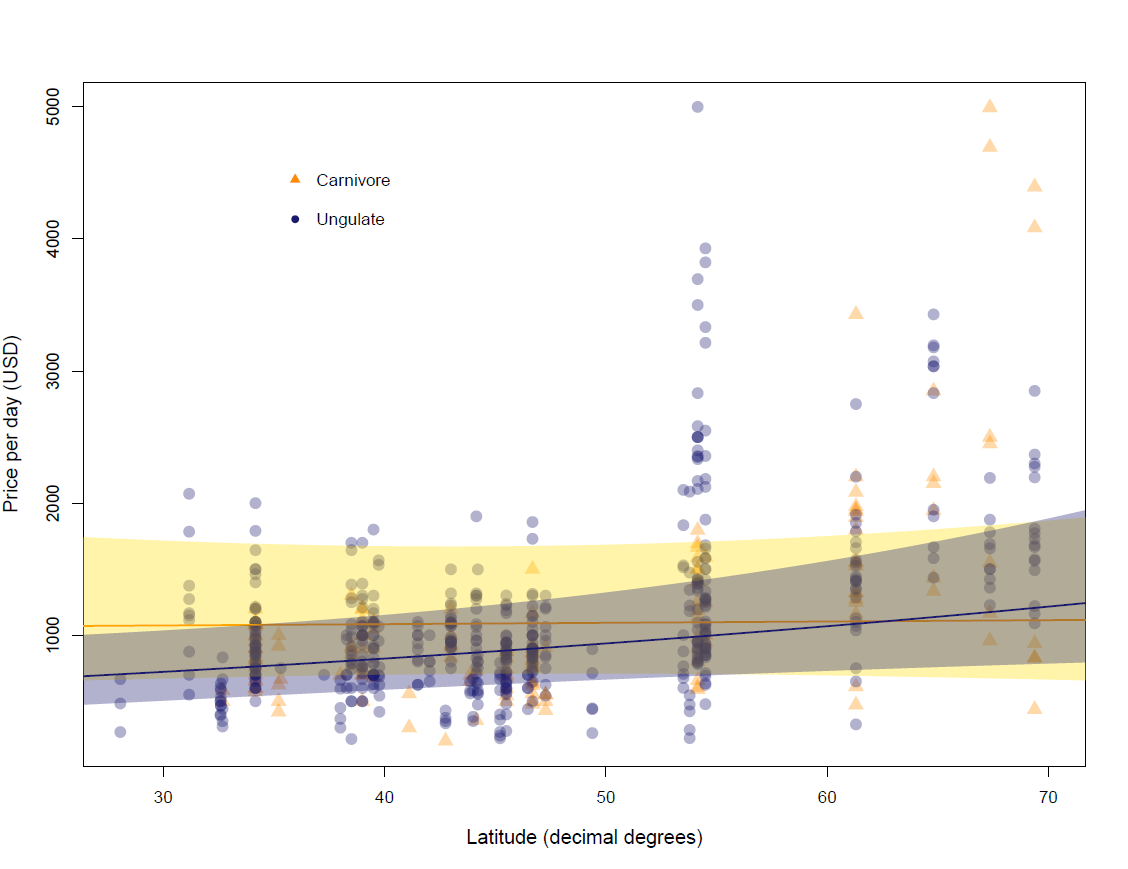

Supplement: Figure S5 from Trophy hunters pay more to target larger-bodied carnivores [file rsos191231supp5.docx]
